# Selective UV Sensing for Energy‐Efficient UV‐A Artificial Synapses Using a ZnO/ZnGa_2_O_4_ Heterojunction Diode

**DOI:** 10.1002/smll.202500098

**Published:** 2025-03-05

**Authors:** Taslim Khan, Santanu Kandar, Sazid Ali, Pushpapraj Singh, Ray‐Hua Horng, Rajendra Singh

**Affiliations:** ^1^ Department of Physics Indian Institute of Technology Delhi New Delhi 110016 India; ^2^ International College of Semiconductor Technology (ICST) National Yang Ming Chiao Tung University Hsinchu 30010 Taiwan; ^3^ Centre for Applied Research in Electronics (CARE) Indian Institute of Technology Delhi New Delhi 110016 India; ^4^ Institute of Electronics National Yang Ming Chiao Tung University Hsinchu 30010 Taiwan; ^5^ Department of Electrical Engineering Indian Institute of Technology Delhi New Delhi 110016 India

**Keywords:** artificial synapse, deep ultraviolet photodetector, heteroepitaxial growth, neuromorphic computing, selective sensing

## Abstract

As neuromorphic computing systems, which allow for parallel data storage and processing with high area and energy efficiency, show great potential for future storage and in‐memory computing technologies. In this article, a high‐performance UV detector for artificial optical synapse applications is demonstrated that can selectively detect UV‐A and UV‐C, with a responsivity of 407 A W^−1^. The pyrophototronic effect increases photocurrent dramatically under UV‐A irradiation due to heat accumulation in the ZnO layer and ZnGa_2_O_4_’s low thermal conductivity. In context of synaptic device, it's shown that a ZnO/ZnGa_2_O_4_ heterostructure can be used as a light‐tunable charge trapping medium to create an electro‐photoactive synapse. The photogating effect enables via pyrophototronic, which traps photogenerated electrons within the ZnO/ZnGa_2_O_4_ interface, and drives synaptic activity, as proven by electrical techniques based on UV‐A stimuli. This phenomenon results in a selective detection capability for UV‐A over UV‐C. Thermally produced pyrophototronic effect synaptic plasticity, simulating biological synapse activity. Persistent photoconductivity under 380 (UV‐A) nm UV light mimics synaptic processes, with low thermal conductivity enhancing synaptic weight updates during learning and forgetting. These findings show the possibility of using ZnO/ZnGa_2_O_4_ heterostructures into artificial optoelectronic synapse systems controlled by thermal dynamics.

## Graphical abstract

The graphical abstract depicts a ZnO/ZnGa_2_O_4_ heterostructure device that demonstrates selective detection of UVA and UVC by the pyrophototronic effect. The device exhibits artificial synaptic behavior by forming the letter “T” with a 25‐pixel array and illustrates synaptic plasticity by the forgetting the “T” over 180 s, emphasizing its learning and forgetting behaviors.

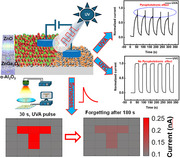

## 1 Introduction

Complex and structured mathematical problems have been addressed for a long time based on the Von Neumann architecture.^[^ ^1^ ^]^ However, as data output increases, this traditional paradigm has limits such as high energy consumption and inefficiency when processing huge amounts of complex information.^[^ ^2^, ^3^, ^4^ ^]^ To address these obstacles, a novel neuromorphic computer architecture inspired by the human brain has been developed to solve complex, unstructured problems using mechanisms such as memory and experience‐based learning.^[^ ^5^, ^6^ ^]^ Synapses are critical components of the brain, allowing for high‐performance data processing and parallel storage. Interestingly, the energy necessary to initiate a biological synaptic event, which normally ranges between 1 and 100 f J, supports cognitive tasks like as learning, picture recognition, and natural language processing.^[^ ^7^, ^8^ ^]^ To meet the growing demand for secure and precise data storage, photo‐memory systems powered by UV light are emerging as a possible alternative. These devices have different advantages than their visible light‐based equivalents, owing to the lower interference from the solar spectrum on Earth. While visible light photo‐memory devices based on materials such as silicon, perovskites, and transition metal dichalcogenides (TMDCs) have been extensively researched, UV‐A based photo‐memory devices have received little attention, despite their potential to meet the growing demand for more secure information storage technologies. Photo‐memory functionalities in UV photodetectors (UV PDs) have enormous potential, especially for applications that require both UV light detection and storing. However, most research efforts have been focused on boosting the response speed of UV PDs, which conflicts with the need for long‐term memory in picture storage applications. Thus, there is an urgent need to build UV PDs with improved photo‐memory features that can keep information for long periods of time.^[^ ^9^, ^10^, ^11^ ^]^

The UV photodetectors that utilize ZnO have attracted a lot of interest because of its effective surface absorption capabilities and wide bandgap.^[^ ^8^ ^]^ However, the presence of an oxygen vacancies on the ZnO surface can cause a delay in response time, frequently in the order of several seconds.^[^ ^12^, ^13^ ^]^ While this delayed response is useful for some photo‐memory applications, it is insufficient for long‐term image storage, necessitating techniques to lengthen the decay time. One interesting method is to create an all‐oxide epitaxial heterostructure by epitaxially growing ZnO on MOCVD‐grown ZnGa_2_O_4_. The low thermal conductivity of ZnGa_2_O_4_ reduces carrier movement and increases memory life by utilizing the pyrophototronic effect, which results from limited heat dissipation in the ZnGa_2_O_4_ layer above sapphire.^[^ ^14^, ^15^ ^]^ The band alignment and crystal structure of ZnGa_2_O_4_ make it an ideal candidate, acting as a barrier that restricts heat flow and photogenerated carrier movement, allowing UV‐A light to keep its memory for longer periods of time.

Beyond ZnO‐based systems, researchers have recently begun investigating various wide bandgap materials for solar‐blind photodetectors, focusing on the UV‐C spectrum (below 280 nm) because to its importance in applications such as missile detection, fire alarms, and environmental monitoring. Materials like Al_x_Ga_1−x_ N, Zn_x_Mg_1−x_O, diamond, and β‐Ga_2_O_3_ are viable options.^[^ ^6^, ^16^, ^17^, ^18^ ^]^ ZnGa_2_O_4_ has emerged as a particularly promising material for solar‐blind photodetectors. ZnGa_2_O_4_ has a large direct bandgap of ≈5.2 eV, allowing detection at wavelengths less than 240 nm, making it ideal for UV‐C photodetection. Furthermore, ZnGa_2_O_4_ is well‐known for its superior crystal quality and potential integration with nanostructured photodetectors, which offer high internal gains and increased detection performance.^[^ ^19^, ^20^ ^]^ Heterostructures can be produced by combining ZnO with ZnGa_2_O_4_, which has a minimum lattice misfit, to improve carrier transport characteristics and UV photodetectors' performance.

In recent research, amorphous ZnGa_2_O_4_/NiO and ZnS/ZnO/ZnS heterostructures have been investigated for UV detection, but the high dark currents induced by defects in the amorphous ZnGa_2_O_4_ limit their practical implementation.^[^ ^8^, ^21^ ^]^ Nonetheless, breakthroughs in nanostructured materials are pushing the sub‐band UV photodetector technology. As in secure UV‐based communication systems, UV‐A/UV‐C wavelengths can convey encrypted signals that are hard to intercept. The system's synaptic‐like activity allows us to replicate adaptive learning or dynamic reaction to external inputs, improving communication security and error correction.^[^ ^22^, ^23^, ^24^ ^]^ So, polarity‐specific UV detection employing plasmonic effects in Ga_2_O_3_ devices with silver nanoparticles has been achieved, enabling for selective detection of UV‐A and UV‐C bands. Similarly, some approaches such as SnS_2_ nanosheet/PbS colloidal quantum dots have demonstrated dual‐response behavior to UV and infrared light, opening new options for innovation in multispectral detection. Above mentioned advancements in amorphous and polycrystalline heterostructures have shown limited performance, particularly at the interfaces, which hinders their effectiveness. While some studies have employed nanoparticles for selective UV band detection, devices based on 2D materials and nanoparticles often suffer from poor repeatability and stability. For industrial applications, there is a pressing need for single‐crystalline semiconductor epitaxy to achieve optimal performance in photodetectors and ensure reliable utilization in artificial synaptic devices.^[^ ^25^, ^26^ ^]^ Single‐crystalline materials offer superior optimization potential, making them more suitable for these advanced applications.^[^ ^15^ ^]^

Building on these advances, the current study seeks to fabricate a single crystalline ZnO/ZnGa_2_O_4_ heterojunction‐based UV photodetector with ultra‐fast switching and selective detection of UV‐A and UV‐C light. To enable selective detection and artificial synaptic functionality, the PDs makes use of the pyrophototronic phenomenon, which modulates thermally induced charge transport by light exposure. This heterostructure allows for fast switching of 0.65 s timescales by leveraging ZnGa_2_O_4_’s sluggish thermal diffusion characteristics and the energy barriers produced by the ZnO. Furthermore, the device's distinct synaptic activity is investigated for energy‐efficient neuromorphic computing applications, revealing the viability of next‐generation UV‐A photodetectors with built‐in memory and learning capabilities.

## 2 Results and Discussion

### 2.1 Structural and Optical Investigations of ZnO/ZnGa_2_O_4_ Heterostructure

**Figure 1:**
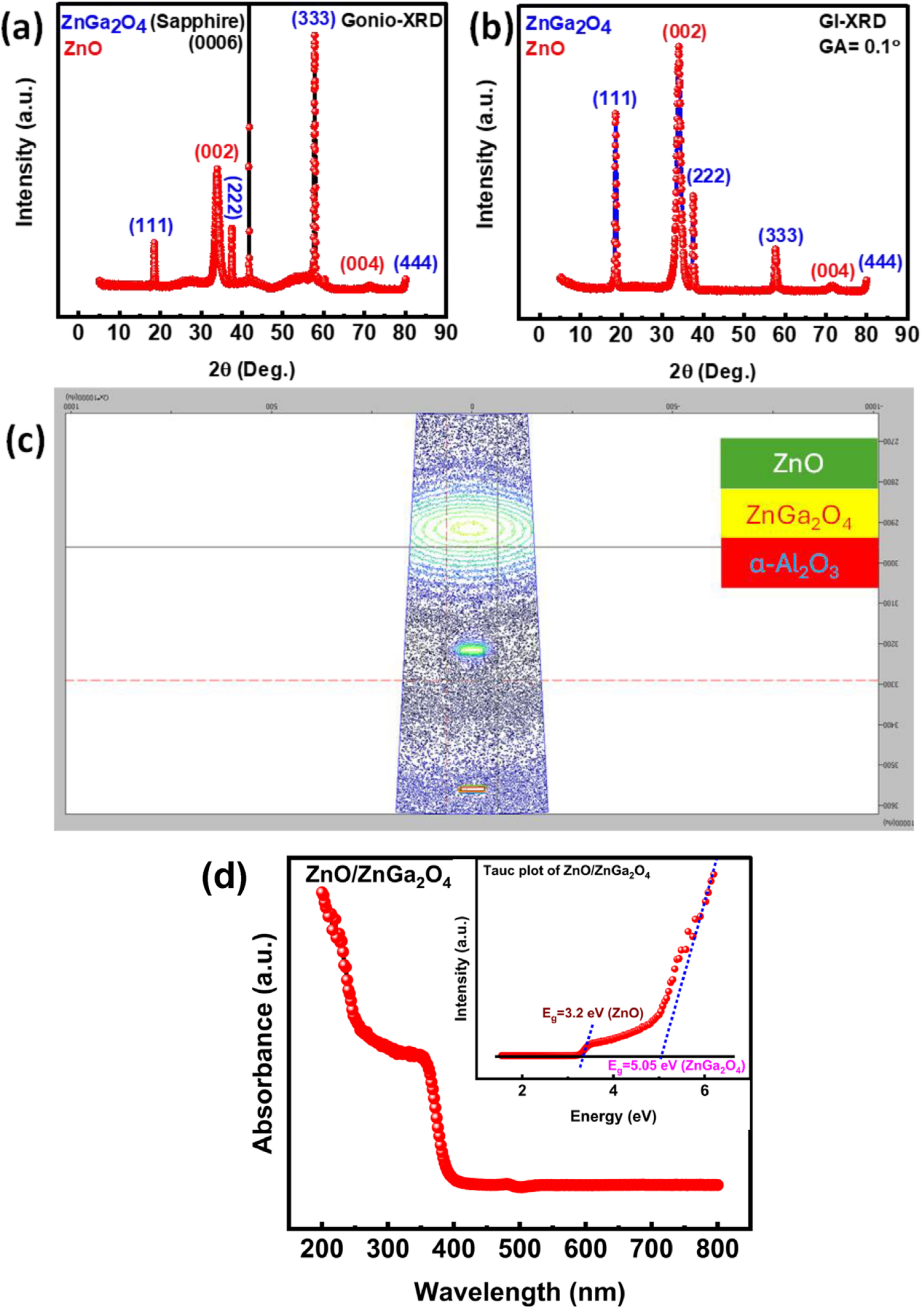
a) Gonio‐XRD of ZnO/ZnGa_2_O_4_ heterostructure on sapphire, b) GI‐XRD of ZnO/ZnGa_2_O_4_ heterostructure to removing out the sapphire effect in XRD, c) reciprocal space mapping of ZnO/ZnGa_2_O_4_ heterostructure on sapphire and d) absorbance spectra of ZnO/ZnGa_2_O_4_ heterostructure with inset tauc plot corresponding the absorbance of ZnO/ZnGa_2_O_4_ heterostructure.

Maintaining the integrity of heteroepitaxial layers requires close attention to the interfacial quality and the layer crystallinity. The sequential development of ZnO layers along the (002) plane is depicted in the high‐resolution X‐ray diffraction (HR‐XRD) plot shown in **Figure** **1**a,b through both mode of XRD Gonio and Grazing incidence (GI). This plane is mirrored in ZnGa_2_O_4_ atop the c‐plane sapphire substrate. This detailed illustration emphasizes how important crystallographic alignment is to attain desired material properties during the integration of epitaxial layers. Through careful examination, including reciprocal space mapping (RSM) along the (0006) plane of the sapphire substrate, the heteroepitaxial development of ZnO/ZnGa_2_O_4_ was confirmed. Parallel planes of ZnGa_2_O_4_ (111) and ZnO (002) were found in the RSM, indicating the intended heteroepitaxial alignment described in Figure 1c. Further optical investigation to calculate the bandgap of integration thin films in heterostructure. The Figure 1d displays the UV–vis absorption spectra of ZnO/ZnGa_2_O_4_ heterostructure on sapphire, revealing distinct absorption edges, ≈250 nm for ZnGa_2_O_4_ and 380 nm for ZnO. Utilizing the tauc plot method^[^ ^27^ ^]^ (Figure 1d) inset), the optical bandgaps were determined as 5.05 eV for ZnGa_2_O_4_ and 3.2 eV for ZnO. These values align with previously reported bandgaps for crystalline ZnO and ZnGa_2_O_4_ films.

### 2.2 XPS Investigations of ZnO/ZnGa_2_O_4_ Heterointerface

**Figure 2:**
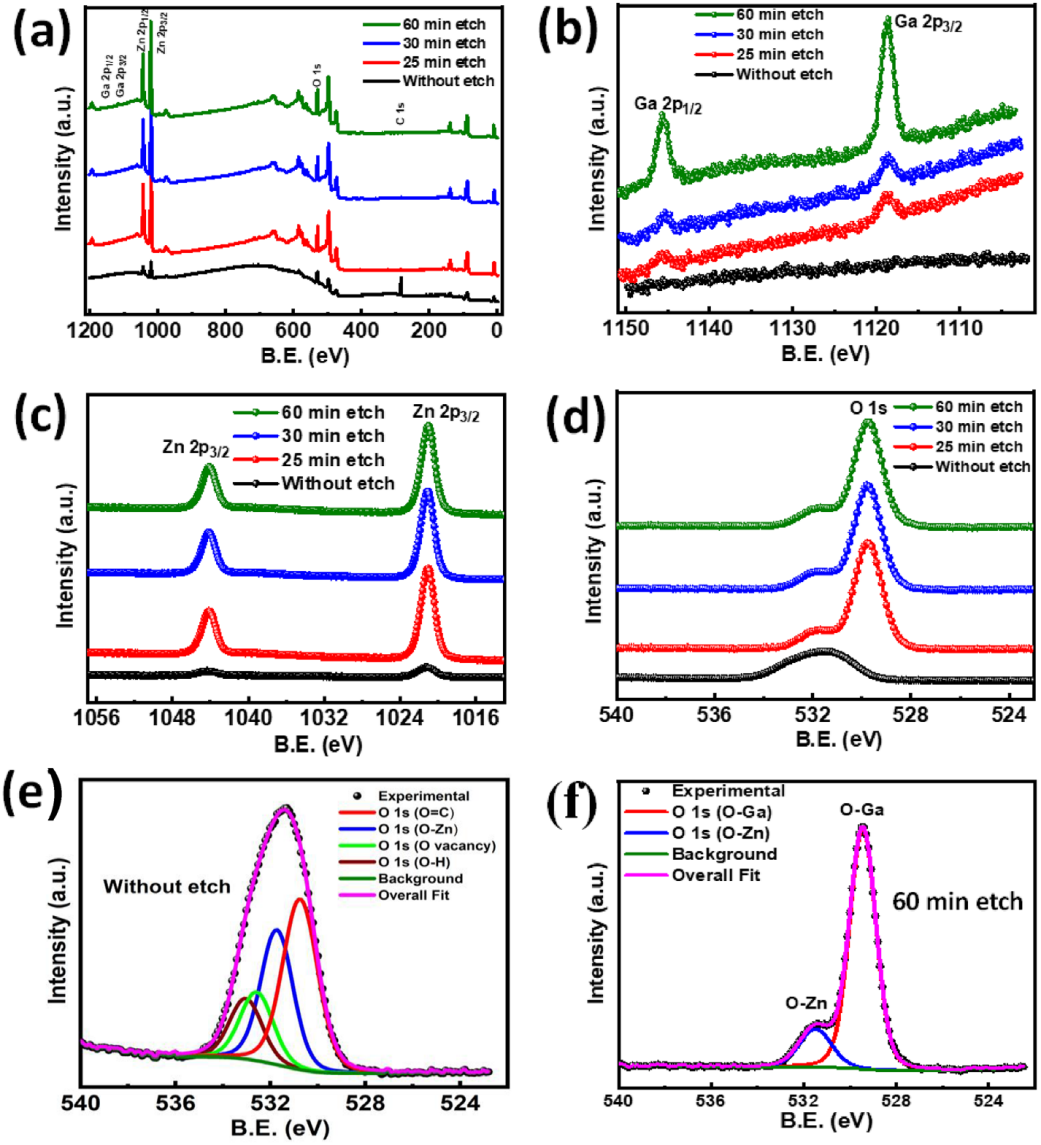
HR‐XPS of ZnO/ZnGa_2_O_4_ of heterostructure a) survey spectra of surface and with different etching time, b) Ga 2p spectra of surface and with different etching time, c) Zn 2p spectra of surface and with different etching time d) O 1s spectra of surface and with different etching time, e) fitted O 1s peak spectra of surface and f) fitted O 1s peak spectra of surface after 60 etching.

**Figure 3:**
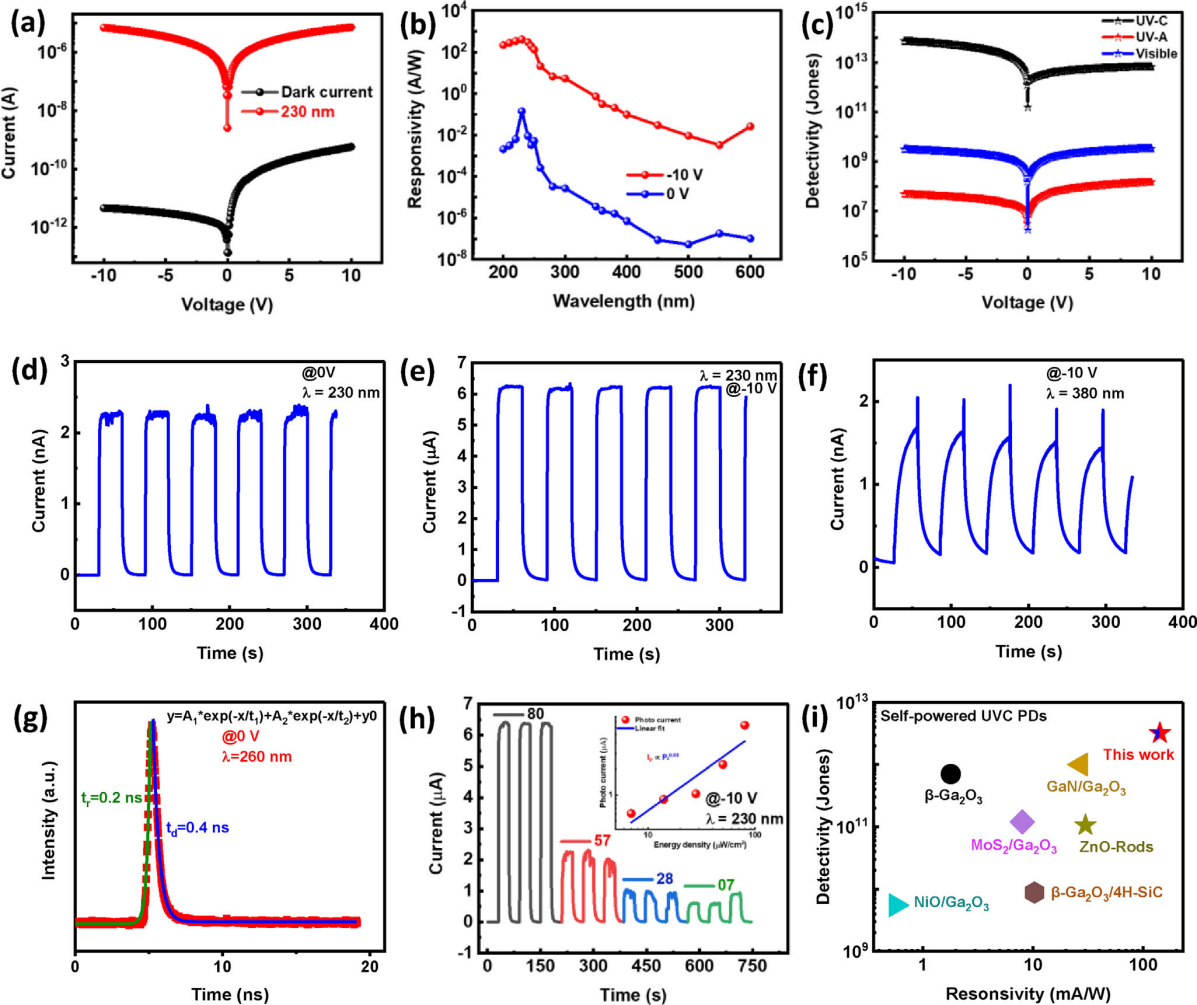
a) dark current and photocurrent variation of −10 to 10 V applied voltage sweep of the heterostructure of the ZnO/ZnGa_2_O_4_ b) spectral response on peak wavelength on applied −10 V and without applied bias and c) detectivity variation for the UVC, UVA and visible region Temporal response of ZnO/ZnGa_2_O_4_ heterostructure device d) self‐powered dynamic response at peak wavelength of 230 nm e) dynamic response at peak wavelength of 230 on applied bias of −10 V f) dynamic response at 380 nm on‐10 V applied bias g) switching speed of the ZnO/ZnGa_2_O_4_ device: ultra‐fast decay and rise time observed 0.2 and 0.4 ns respectively h) dynamic temporal response over varying the energy density of peak wavelength of 230 nm and i) performance comparison of reported self‐powered UVC (230 nm) with ZnO/ZnGa_2_O_4_ heterostructure based UVC self‐powered photodetector on basis of responsivity and detectivity.

The ZnO/ZnGa_2_O_4_ heterostructure was characterized using Kratos Analytical AXIS Supra X‐ray photoelectron spectroscopy (XPS) to detect the presence of elements on the film's surface and within the depth of the heterostructure. Ar+ ion beam sputtering was employed to analyze the elements at different depths of the film by varying the etching time. The energy of the Ar^+^ ion etching was 5 keV and the etching time was varied from 0 to 60 min. **Figure** **2**a shows the XPS Survey spectra of ZnO/ZnGa_2_O_4_ heterostructures with different etching times, illustrating the changes in different elements (Ga, Zn, O, and C) with etching time. The C 1s signal, indicating surface contamination, decreases with etching time and disappears after 60 min of sputtering. Figure 2b shows the Ga 2p core level spectra for different etching times. Initially, there is no signal from Ga 2p at the surface of the heterostructure. However, as the etching time increases from 0 to 60 min, the Ga 2p signal intensity also increases and observes an intense peak after 60 min of etching. Ga 2p_3/2_ and Ga 2p_1/2_ peak positions are found to be 1118.6 and 1145.6 eV, respectively which matches the reported peaks. **Figure** **3**c presents the core level spectra of Zn with the etching time. The Zn 2p signal is detected throughout the depth, with an increase in intensity as the etching time increases. As Ar+ ion etching does not provide a sharp edge, the Zn signal also originates from the top ZnO layer, contributing to the Zn 2p signal. As a result, both the signal from ZnGa_2_O_4_ and ZnO increases the Zn 2p signal intensity. The Zn 2p_3/2_ and Zn 2p_1/2_ peak positions are found as 1021.0 and 1044.1 eV, respectively, matching the literature results. Figure 2d displays the O 1s spectra with varying etching times. As the etching time increases from 0 to 60 min, the oxygen signal from ZnGa_2_O_4_ and the sapphire substrate appears in the spectra, increasing the intensity of the oxygen spectra. The O 1s core level spectra of 60 min of etching and without etching were fitted using CasaXPS software with a Shirley background depicted in Figure 2e. At the heterostructure surface, O 1s exhibits three peaks at 530.8, 531.8, and 533 eV, corresponding to O═C, O─Zn, and O─H bonds, respectively. In addition to these three peaks, O 1s shows an oxygen vacancy‐related state of ≈232.5 eV in the O 1s spectra.^[^ ^28^ ^]^ Figure 2f describes, after 60 min of etching, the O 1s spectra only show peaks corresponding to O─Zn and O─Ga bonds at 531.8 and 528.4 eV, respectively. The variation of the elements with etching time indicates that the carbon and hydrogen contamination present in the top surface of the heterostructures vanishes after sputtering. Also, indicates that the O─Ga bond is not present on the top surface, and appears only after the sputtering.

### 2.3 Self‐Powered Deep UV Photodetector

The fabricated self‐powered photodetector with a ZnO/ZnGa_2_O_4_ heterostructure and conducted a systematic analysis of its optoelectronic performance. The results, as shown in the Figure 3a, demonstrate the device's very low dark current under different bias. At −10 V, the dark current was just 4.49 *p*A, and it dropped even lower to a remarkable 132 *f*A at 0 V. Low dark current levels indicate less noise and leakage in the photodetector, which is crucial for achieving high sensitivity for DUV detection. This is especially important for photodetectors that operate in self‐powered or low‐biased modes, as low dark current improves the signal‐to‐noise ratio, hence increasing detection accuracy. Figure 3b depicts the photodetector's spectral responsivity, which highlights the device's excellent performance. It has a peak responsivity of 407 AW^−1^ at 230 nm at −10 V applied bias, which is also found as 142 mAW^−1^ with applied voltage calculated by the mathematical expression^[^ ^29^ ^]^ as $R_{\lambda }=\frac{I_{p}-I_{d}}{P_{\lambda }D}\;$, here ***I_p_*** is photocurrent ***I_d_*** stands for dark current, *P*_λ_ referred for the power density and ***D*** is the active area of the device. Compared to traditional DUV photodetectors, which frequently exhibit substantially lower responsivity, this ZnO/ZnGa_2_O_4_ PDs stands out for its efficient light absorption and charge separation capabilities. This is most likely due to the perfect band alignment of ZnO and ZnGa_2_O_4_, which facilitates effective charge transfer while minimizing recombination losses. The fact that such high responsivity is attained in self‐powered settings emphasizes the device's potential for energy‐efficient optoelectronic applications.^[^ ^6^, ^30^, ^31^ ^]^

Furthermore, the photodetector exhibits a remarkable visible to UV‐C rejection ratio reaching −10^5^, among the highest known values. This demonstrates its capacity to effectively distinguish between visible and DUV radiation, making it extremely selective and appropriate for solar‐blind applications. Figure 3c shows detectivity values for UVC, UVA, and visible regimes calculated by the mathematical expression^[^ ^29^ ^]^ as D^*^ $=\sqrt{\frac{D}{2eI_{d}}}\;R_{\lambda }$ jones. UV‐C detectivity can reach 7.23 × 10^13^ Jones. This strong detectivity, along with a low dark current, makes the device appropriate for applications requiring weak UV signal detection. Figure 3d,e demonstrate the dynamic temporal response under ON/OFF of light with 30 s interval, with the device exhibiting quick and stable responses to 230 nm illumination at −10 V, as well as consistent and repeatable behavior in self‐powered mode. This fast response time is critical for high‐performance photodetectors that require rapid signal processing. Notably, Figure 3f depicts the dynamic temporal response in the UVA region at 380 nm, where a significant spike is detected in the off state due to the pyrophototronic effect. This effect, mediated by the interplay of pyroelectric polarization with photogenerated carriers, improves the device's responsiveness to temperature fluctuations. The reproducibility of this effect across 30 cycles demonstrates the device's robustness. To assess the PDs' switching speed, simply assess the device's suitability for the specific application of deep UV sensing. The ZnO/ZnGa₂O₄ heterostructure PDs transient photoresponse was tested using a Horiba of the Deltadiode 260 nm solid‐state diode laser and a with TRPL system^[^ ^32^ ^]^ depicted in Figure 3g Fitting the transient response curves with bi‐exponential equations of rise time calculations, $I=I_{o}+K_{1}e^{\frac{t}{\tau_{r1}}}+K_{2}e^{\frac{t}{\tau_{r2}}}$ and decay time calculations $I=I_{o}+K_{3}e^{-\frac{t}{\tau_{d1}}}+K_{4}e^{-\frac{t}{\tau_{d2}}}$ here, *I_o_* stands for the steady state current, K_1_, K_2_, K_3_, K_4_ are the empirical constant τ_*r*1_ rise time and τ_*d*1_ decay time.^[^ ^33^, ^34^, ^35^ ^]^ Yielded rise and decay periods of 0.2 and 0.4 ns, respectively. Further, to investigate the actual speed of ZnO/ZnGa_2_O_4_ heterostructure photodetector at 230 nm with a −10 V bias has a rise time of 0.65 s and a decay time of 0.9 s, indicating its speed. Transient characteristics are presented in, Figure S6 (Supporting Information). The ZnO/ZnGa_2_O_4_ heterostructure photodetector uses strong avalanche electric fields to separate and transport electrons, minimizing carrier aggregation at the interface. Compared to mature Si‐or InP‐based photodetectors, this device has one of the fastest response speeds among ZnGa_2_O_4_‐based photodetectors so far.^[^ ^34^, ^36^, ^37^, ^38^ ^]^ This performance confirms the device's suitability for fast, reliable UV‐C sensing. Figure 3h displays the I‐T response across varying energy densities from 80 to 7 µW cm^−^^2^, showcasing the device's repeatability and stability. The inset of Figure 3h provides insight into the device's linearity by illustrating the relationship between the photoexcited current and power densities, modeled with the power law  *I_P_* =  *kP*_λ_^*q*^. Here, the exponent **q**, a key indicator of the current transport mechanism, is found to be 0.88—close to unity—suggesting minimal defects between the Fermi level and the conduction band. This near‐unity **q**‐value points toward a gain mechanism, further enhancing the device's high‐performance photodetection capabilities. Figure 3i shows the ZnO/ZnGa_2_O_4_ heterostructure‐based self‐powered UVC photodetector outperforms prior self‐powered UVC (230 nm) devices, displaying higher responsivity and detectivity. Its improved performance demonstrates advances in sensitivity and reliability, establishing it as a highly efficient UVC detection alternative.^[^ ^31^, ^39^, ^40^, ^41^, ^42^, ^43^, ^44^ ^]^ Overall, the photodetector has outstanding performance metrics, making it an attractive choice for DUV detection, artificial synapse applications, and energy‐efficient sensor networks. The stability of the device after the 5 months is depicted in the Figure S7 (Supporting Information).

### 2.4 Mechanism of Selective UVC and UVA Responses

This ZnO/ZnGa_2_O_4_ heterostructure PDs that can selectively detect UVA (380 nm) and UVC (230 nm) light via the pyrophototronic effect. The effect relies on thermal‐induced dipole reorientation within the material. ZnO and ZnGa_2_O_4_ have unique temperature responses and dipole dynamics of their non centrosymmetric crystal lattice structure and very low thermal conductivity of 22.9 W mK⁻¹, enabling the device to distinguish between UV‐A and UV‐C irradiation.^[^ ^45^, ^46^ ^]^ Also, device's sensitivity and memory function for UV‐A light are a result of the dissociation of adsorbed oxygen on the ZnO surface and the generation of photogenerated carriers, which modify the depletion region under UV‐A exposure. As seen in Figure 6b, UV‐A irradiation causes the separation of holes and electrons in ZnO, resulting in photogenerated carriers. Photo‐induced holes move to the surface contact and react with oxygen (O_2_), releasing adsorbed oxygen (O_2_ (ad) + h → O_2_(g)). As a result, the depletion zone at the interface narrows, lowering interface resistance.^[^ ^47^, ^48^ ^]^ The photogenerated electrons that remain improve the ZnO epilayer's conductivity, increasing current flow through the device and allowing UV‐A detection. The device's memory of UV‐A exposure is based on the progressive re‐adsorption of oxygen. When UV‐A light is withdrawn, oxygen re‐adsorption slows and gradually decreases the current. This response is highly dependent on the oxygen content in the surrounding environment.^[^ ^49^, ^50^ ^]^

**Figure 4:**
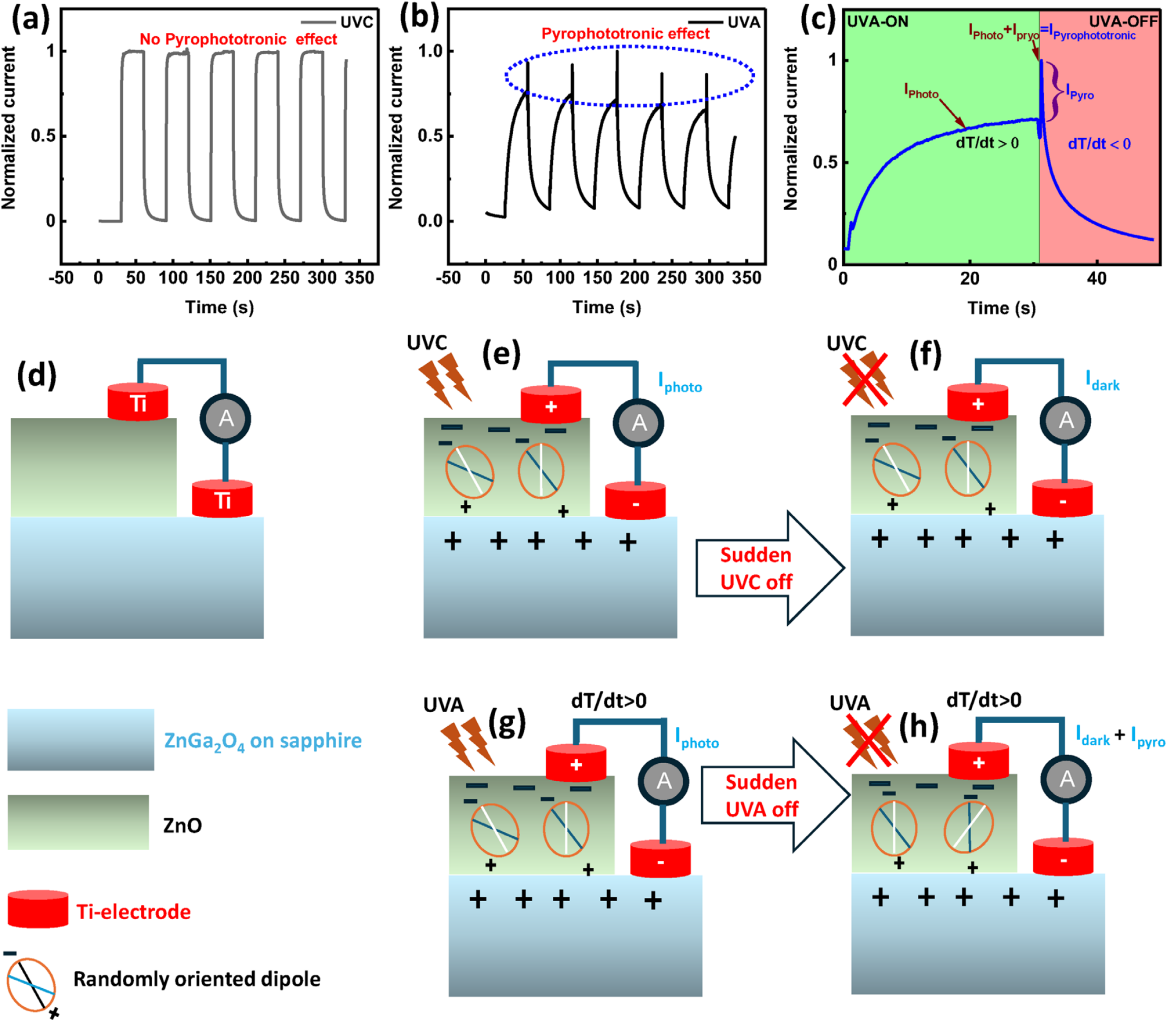
a) Dynamic temporal response in UVC without pyrophototronic current spikes b) dynamic temporal response in UVA with pyrophototronic current spikes, making the device a selective for the UVA through pyrophotronic effect, c) magnified view of the single cycle temporal response to better understanding of pyrophotronic effect in device, d) a schematic of heterostructure, e) UVC illumination on the ZnO/ZnGa_2_O_4_ heterostructure diode major absorption along ZnGa_2_O_4_, f) no‐Illumination of UVC on the ZnO/ZnGa_2_O_4_ heterostructure, g) UVA illumination on the ZnO/ZnGa_2_O_4_ heterostructure diode major absorption along ZnO, and h) No UVA illumination on the ZnO/ZnGa_2_O_4_ resulted polarization of carriers occurring enhance the current, slows down the speed.

This study investigates the sources of these selective reactions and includes extensive figures that depict the device's dynamic temporal response, structure, and detecting processes. The ZnO/ZnGa_2_O_4_ PDs exhibits distinct responses to UV‐A and UV‐C wavelengths. **Figure** **4**a depicts the temporal response under UV‐C illumination, which is constant and absent of spikes. However, as illustrated in Figure 4b, the reaction to UV‐A is characterized by strong pyrophototronic current spikes, a phenomenon caused by the pyrophototronic effect that allows the device to respond selectively to UV‐A light. Figure 4c depicts zoomed view of a single cycle of the temporal response, shedding light on current spike generation and the impact of dipole reorientation on performance. Understanding the wavelength‐selective response of the ZnO/ZnGa_2_O_4_ heterostructure depends on its integrated oxide layers as shown in Figure 4d. Figure 4e shows that UV‐C light mostly absorbs in the ZnGa_2_O_4_ layer, resulting in low thermal disturbance and a spike‐free response. Figure 4f shows that there is no photogenerated current when UV‐C illumination is not present. Under UV‐A light, absorption shifts to the ZnO layer, as seen in Figure 4g, resulting in a significant pyrophototronic response characterized by current spikes. This reaction fades when UV‐A illumination is turned off, as shown in Figure 4h, demonstrating the device's selectivity.

The ZnO/ZnGa_2_O_4_ device's selectivity depends on the pyrophototronic effect, which is regulated by thermal gradients (dT/dt) and dipole orientation dynamics within the ZnO layer. When UV‐A light strikes ZnO, it is quickly absorbed, creating a temperature increase that results in a positive dT/dt. This rapid heat accumulation causes random dipole orientation in the ZnO layer, increasing the system's entropy as considering the dipole is the microstate (Ω) of the system as per the (Equations 1 and 2). Entropy of dipole orientation, arrangement microstates consisting the dipole

(1) $$S=\int \frac{\Delta Q}{T}$$

(2) $$S=k_{b}\;\ln\Omega$$

where S is the entropy of the combined heterostructure on the illumination, Ω is dipole microstates, Δ*Q* is change in energy given to system by the illumination, and T temperature at specific moment when the sharply heat change occurs. During this time, the device's response slows as it approaches a high‐entropy state, with photogenerated holes accumulating at oxygen vacancies, facilitating the production of free electrons in ZnO's conduction band and increasing conductivity under UV‐A illumination. When UV‐A light is turned off, the temperature gradient reverses (dT/dt becomes negative), resulting in a cooling phase that favors ordered dipole reorientation as entropy diminishes. When the system returns to ordered dipole alignment, there is a dramatic current spike as it stabilizes. The pyrophototronic effect, represented by the spike in Figure 4b, does not occur under UV‐C light due to major absorption in the ZnGa_2_O_4_ layer of the ZnO/ZnGa_2_O_4_ layer, which prevents dipole reorientation and entropy fluctuations. ZnO/ZnGa_2_O_4_ has low thermal conductivity, allowing heat from UV‐A absorption to escape slowly. This creates a temperature gradient that increases the duration of dipole reorientation within ZnO. This property allows for continuous current spikes specific to UV‐A detection, whereas the steady response to UV‐C lacks such spikes, aiding in the discrimination of the two wavelengths. ZnGa₂O₄’s limited heat conductivity sustains the pyrophototronic action within ZnO, allowing selective UV‐A detection while reducing responses to UV‐C. The ZnO/ZnGa_2_O_4_ heterostructure's selective detection capacity is based on the unique optical and thermal properties of ZnO and ZnO/ZnGa_2_O_4_. This PDs can distinguish between UV‐A and UVC wavelengths with high accuracy thanks to the pyrophototronic effect. UV‐C detection relies on focused absorption in the ZnGa_2_O_4_ layer, which prevents the pyrophototronic effect. In contrast, UV‐A illumination causes absorption in ZnO, resulting in dipole reorientation and significant current spikes when the illumination is turned off. The ZnO/ZnGa_2_O_4_ heterostructure is ideal for wavelength‐specific detection in applications like energy‐efficient artificial synaptic devices and improved photodetectors with selective UV sensing. The ZnO/ZnGa_2_O_4_ heterostructure photodetector effectively detects UV‐A over UV‐C, driven by the pyrophototronic effect. Under UV‐A illumination, thermal gradients, and dipole reorientation within ZnO contribute to the selective response. The poor thermal conductivity of ZnGa_2_O_4_ allows for prolonged current spikes to differentiate between UV‐A and UV‐C.

### 2.5 Pyro‐Phototropic Effect Modulated UVA Artificial Synapse

**Figure 5:**
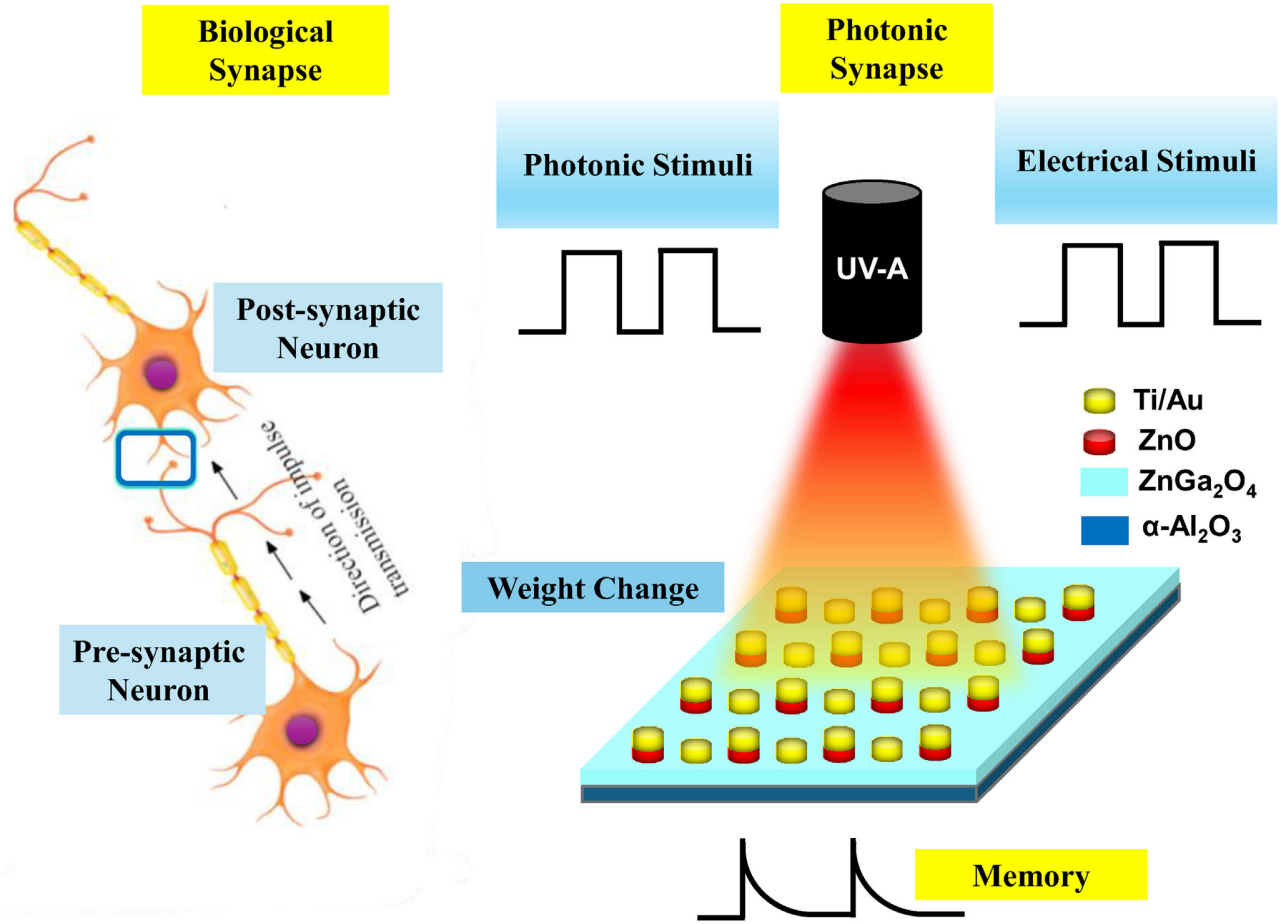
Schematic illustrating biological synaptic behavior alongside a photonic synaptic device modulated by optical stimuli.

**Figure 6:**
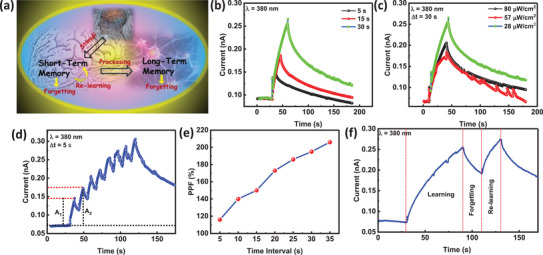
a) Schematic depicting the memory process in the human brain b)I‐T on the different, c) I‐T on different UVA light intensities on −10 V applied bias, d) Current modulation in the ZnO/ZnGa_2_O_4_ heterostructure caused by six successive light pulses (80 µW cm^−^^2^, applied bias = −10 V) e) A_1_ and A_2_ denote the amplitude of the current change during the initial and subsequent light pulses of the 5 s photonic paired pulse facilitation (PPF) index in relation to the time interval between consecutive pulses f) measured learning and relearning experience behavior of the optical synaptic device just on 80 µW cm^−^^2^ pulse intensity

The retina converts the image information captured by the eyes into neural signals, which are then transmitted to the brain for storage. This process is facilitated by visible light. However, human visual memory mechanisms are unable to perceive or remember UV region information. To enable UV light sensing and memory, we created an artificial retina made up of an array of UV optoelectronic synaptic devices that can perceive UV light, transmit signals, and perform short‐/long‐term memory tasks (Figure 6a). **Figure** **5** shows, that to simulate pyrophototronic‐driven synaptic behavior and create an optoelectronic synapse activated by UVA light, we used a ZnO/ZnGa_2_O_4_ heterostructure as the artificial retina unit's core.

**Figure 7:**
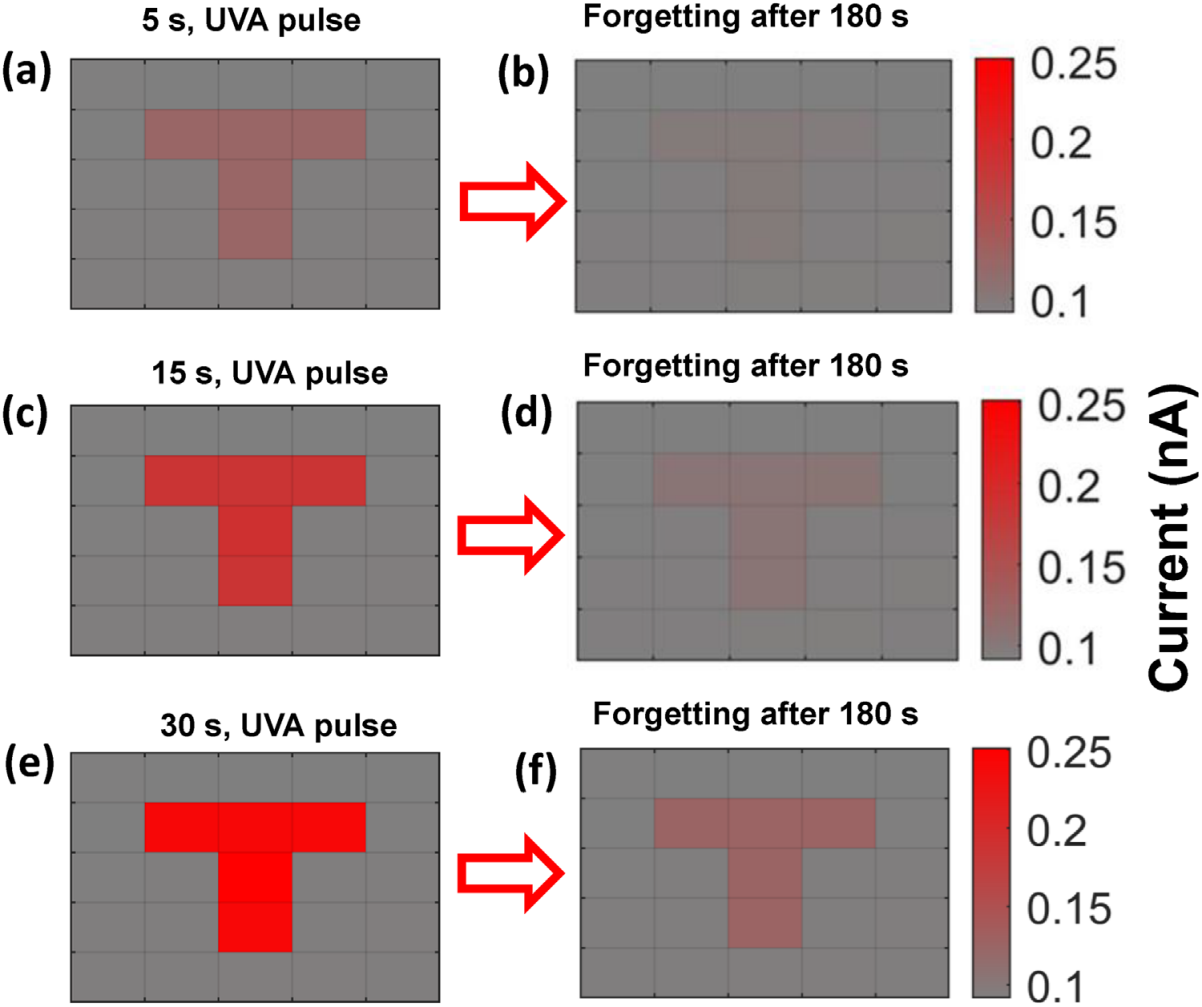
a–f) “T” image learning and the forgetting for the different pulse of the time on fixed energy density of UVA at −10 V of applied bias.

Because of the photonic non‐volatility of ZnO/ZnGa_2_O_4_ heterojunction PDs, it was used to simulate synaptic activities in the human brain. In biological synapses, paired‐pulse facilitation (PPF) is an important phenomenon for decoding temporal information, in which the postsynaptic current (PSC) is increased by successive presynaptic stimulations. **Figure** **6**b,c shows that increasing the UV pulse intensity from 80 to 28 µW cm^−^^2^ led to increased photocurrent and retention, indicating STM‐to‐LTM conversion.^[^ ^8^, ^50^ ^]^ UV pulse duration had a similar effect. Figure 6d shows that the second pulse (A_2_) has a higher PSC than the first pulse (A_1_), with pulse and interval periods of 5 s each.^[^ ^5^, ^7^, ^9^, ^50^ ^]^ This enhancement occurs when photo‐generated carriers do not fully decay to baseline levels before the next pulse is administered, resulting in carrier buildup with subsequent light pulses. Figure 6e shows the PPF ratio as a function of light pulse time (which ranges from 5 to 30 s). At a 30 s interval, the PPF ratio was 204%. In human cognition, signal stimulation that is prolonged or of greater frequency allows the transition from short‐term memory (STM) to long‐term memory (LTM). STM allows for the short‐term storage and manipulation of restricted information, but if neuronal connections are not strengthened, the information goes away. LTM, on the other hand, allows for the permanent retention of information, allowing the recall of previous experiences, knowledge, and abilities. Also, this ZnO/ZnGa_2_O_4_ heterostructure's low operating voltage and strong pyrophototronic effect result in less energy‐intensive synaptic events compared to traditional optoelectronic synapses. Many optoelectronic synapses operate in the pJ to nJ range, while biological synapses consume 1–10 f J for each synaptic event. (Note S1, Supporting Information). The ZnO/ZnGa_2_O_4_ UV PD supports this shift by changing the intensity and duration of UV pulses, which indicate learning strength and time. Furthermore, Figure 6f shows that people learn, forget, and relearn to acquire long‐term memory, with relearning usually taking less time than initial learning. The device replicates this behavior: after a 60 s learning phase and a 20 s forgetting period, the current recovers to its prior level in under 16 s. thus ZnO/ZnGa_2_O_4_ PDs be applied in the field of UV image sensing and storage. Furthermore, our device demonstrates high repeatability, as shown in **Figure** **7** through the image learning ability of the device.^[^ ^6^, ^51^ ^]^

A 5 × 5 photodetector array, shown in Figure 7a–f, demonstrates the “learning experience” and UV image storing capabilities of the ZnO/ZnGa_2_O_4_ heterostructure. Figure 5b shows a “L”‐shaped photomask positioned between the photo‐memory array and the UV light source (380 nm). After 5, 15, and 30 s of UV light fiber‐based source of UV‐A (1 mm spot size) are moving in T shaped considering as a photomask respectively, the photocurrent variation of each individual pixel is measured sequentially by scanning the array. The probe and detector array generate a “T”‐shaped pattern with alternating bright and dark contrast on the photodetector array in Figure S5 (Supporting Information) schematic representation. However, after 180 s of turning off the UV light, the pattern becomes too weak to detect. When the ultraviolet light exposure is increased to 30 s, the bright‐dark contrast becomes more noticeable, and the array preserves the image even after a 180 s “forgetting” period. This behavior is like that of the human brain, in which longer learning periods result in clearer and more durable memories. These findings highlight the UV photodetector's potential for use in UV optoelectronic imaging memory.

## 3 Conclusion

A high‐performance ZnO/ZnGa_2_O_4_ heterojunction photodetector, which has been specifically designed to function as an artificial optical synapse capable of selective UV‐A and UV‐C detection, is presented in this study. This device uses the pyrophototronic effect to improve charge transport via heat‐induced modulation, resulting in outstanding responsivity (407 A W^−1^) and rapid switching (0.4 ns) in the UV‐C region. Under UV‐A light, thermal buildup in the ZnO layer, along with ZnGa_2_O_4_’s low thermal conductivity, enables energy‐efficient neuromorphic functionalities by increasing photocurrent. These thermal dynamics provide memory capabilities akin to biological synapses, paving the way for energy‐efficient synaptic devices and enabling sophisticated neuromorphic computing applications.

To simulate neuroplasticity, the photodetector acts as an electro‐photoactive synapse with both volatile and non‐volatile states, effectively modeling short‐ and long‐term memory processes. The light‐driven charge trapping increases persistent photoconductivity, which is required for parallel data storage and in‐memory computing. The PDs modifies synaptic weight using UV‐A (380 nm) radiation, capturing synaptic behavior for purposes such as learning and forgetting.

Successfully demonstrated the learning and forgetting experience of synaptic device based on sin crystalline ZnO/ZnGa_2_O_4_ heterostructure photodiode. The device exhibits improved stability and repeatability by utilizing single‐crystalline ZnO/ZnGa_2_O_4_ epitaxial materials, which are critical for scaling in industrial applications. This research emphasizes the ZnO/ZnGa_2_O_4_ heterojunction's promise as a next‐generation, UV‐A responsive synaptic device, paving the way for dependable, scalable, and energy‐efficient artificial synapse technology.

## 4 Experimental Section

### Fabrication of ZnO/ZnGa_2_O_4_ Heterojunction Device

The better ZnGa_2_O_4_ epilayers were grown on c‐plane (0001) sapphire substrates using MOCVD, triethylgallium (TEGa), diethylzinc (DEZn), and oxygen (O_2_) as precursors for Ga, Zn, and O, respectively. The corresponding molar fluxes of the TEGa, DEZn, and O_2_ precursors were 4.3 × 10^−5^, 3.3 × 10^−5^, and 3.3 × 10^−2^ mol min^−1^, respectively. For MOCVD, the main flow was N_2_, while for TEGa and DEZn, the carrier gas was high‐purity Ar. The growth pressure was 25 Torr, and the temperature was 720 °C. It took 30 min for the ZnGa_2_O_4_ epilayer to grow to a thickness of 115 nm. Selective area deposition of ZnO was done utilizing a tin physical mask coupled with Kapton tape as a precise means of fabrication. The ZnO epitaxial layer was meticulously deposited onto a ZnGa_2_O_4_ epilayer atop sapphire via RF‐magnetron sputtering employing a 99.99% pure ZnO ceramic target a 3‐inch diameter. Operating at an RF power of 150 watts and maintaining a target‐to‐substrate distance of 9.5 cm, a uniform 350 nm thick ZnO film was achieved. This deposition occurred within a controlled environment, with a base pressure of 3.5 × 10^−6^ mbar and a working pressure of 9.3 × 10^−3^ mbar, ensuring optimal conditions for film growth. Notably, the substrate temperature remained at room temperature (300 K), complemented by the target‐to‐substrate distance of 9.5 cm, ensuring precise and reproducible results. Further, Kapton tap on physical tin metal mask was removed. Then heterostructure were coated with metal contacts (for both Au/Ti of 40/80 nm) using an E‐Gun Evaporation of Auto 500 Vacuum Coating System. For improved metal electrode adhesion, Figure S1 (Supporting Information) presents the fabrication process of the heterointerface in a comprehensive manner.

### Characterization and Photoelectrical Measurements

The crystal structure of ZnO/ZnGa_2_O_4_’s was examined using a Panalytical Empyrean X‐ray Diffraction (XRD) apparatus. Bruker Dimension Icon Atomic Force Microscopy (AFM) pictures were used to record the homogeneity of the as‐grown film before and after irradiation. An ex situ micro‐Raman spectroscope (Horiba Scientific, Lab RAMHR Evolution) with a 250 nm excitation source was used to perform the TRPL measurements at room temperature. The Perkin Elmer, Singapore system was used to obtain the UV–vis spectra. A Kratos Axis Ultra^DLD^ system was used to record X‐ray photoelectron spectroscopy to comprehend in depth quality of heterojunction. To prevent visible light noise, photoelectrical characterizations were carried out on the Keithley SCS‐4200 system in conjunction with a Bentham monochromator (BenthamTMC‐300 V) that had Xenon lamps inside a dark cabinet.

## Conflict of Interest

The authors declare no conflict of interest.

## Supporting information



Supporting Information

## Data Availability

The data that support the findings of this study are available on request from the corresponding author. The data are not publicly available due to privacy or ethical restrictions.
