# Supplementary material for: Selective UV Sensing for Energy‐Efficient UV‐A Artificial Synapses Using a ZnO/ZnGa2O4 Heterojunction Diode
Source: Small. 2025 Mar 5;21(16):2500098. doi: 10.1002/smll.202500098 (PMC12019913; doi:10.1002/smll.202500098)
Supplement: Supplementary file 1 — Supporting Information [file SMLL-21-2500098-s001.docx]

**Supporting information**

**Selective Ultraviolet Sensing for Energy-Efficient UV-A Artificial Synapses Using a ZnO/ZnGa_2_O_4_ Heterojunction Diode**

*Taslim Khan^a, d*^, Santanu Kandar^a^, Sazid Ali^c^, Pushpraj Singh^c^, Ray-Hua Horng^d,e*^ and Rajendra Singh^a, b*^*

*^a^Department of physics, Indian Institute of Technology Delhi, New Delhi, 110016, India*

*^b^Department of Electrical Engineering, Indian Institute of Technology Delhi, New Delhi, 110016, India*

*^c^Centre for Applied Research in Electronics (CARE), Indian Institute of Technology Delhi, New Delhi, 110016, India*

*^d^International College of Semiconductor Technology (ICST), National Yang Ming Chiao Tung University, Hsinchu, 30010, Taiwan*

*^e^Institute of Electronics, National Yang Ming Chiao Tung University, Hsinchu, 30010, Taiwan*

^*^Corresponding Author: [taslimkhan3858@gmail.com](mailto:taslimkhan3858@gmail.com), r[ayhua@nycu.edu.tw](mailto:ayhua@nycu.edu.tw) and [rsingh@physics.iitd.ac.in](mailto:rsingh@physics.iitd.ac.in),

s

**Figure S1.** schematic of the device fabrication (a) a metal mask with Kapton tap on ZnGa_2_O_4_ epilayers on sapphire substrate (b) selected area deposition of ZnO through the RF-sputtering (c)electrode fabrication of Ti/Au of 20/80 nm removing the Kapton tap so that symmetrical electrode can be formed on heterostructure (d) fabricated ZnO/ZnGa_2_O_4_ heterostructure photodetector.

***
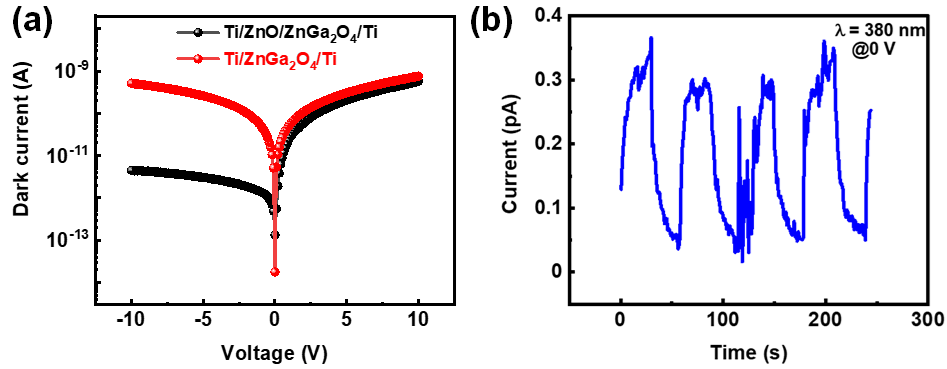
Figure S2.*** *(a) illustration of dark current of ZnO/ZnGa_2_O_4_ heterostructure and ZnGa_2_O_4_ with Ti/Au electrodes to confirm the heterostructure formation and (b) self-powered temporal response for the UVA wavelength.*

**Figure S3.** Atomic force microscopy (AFM) images of the ZnO and ZnGa_2_O_4_ epilayers with 13 nm and 4 nm rms roughness respectively.

**Figure S4** (a) and (b) display the valence band spectra of ZnGa_2_O_4_ (ZGO) and ZnO, respectively, with valence band maxima at 2.98 eV for ZGO and 1.76 eV for ZnO. Figure 1(c) illustrates the band alignment between ZGO and ZnO, determined using the bandgap energy, valence band maximum, and Zn 2p_3/2_ core level binding energy. The band alignment analysis reveals that both ZGO and ZnO exhibit n-type characteristics and that they form a type I band alignment. The calculated conduction band offset (CBO) and valence band offset (VBO) are 0.63 eV and 1.22 eV, respectively.

**Figure S5.** Illustrations of the formation of image of the PDs arrays

**
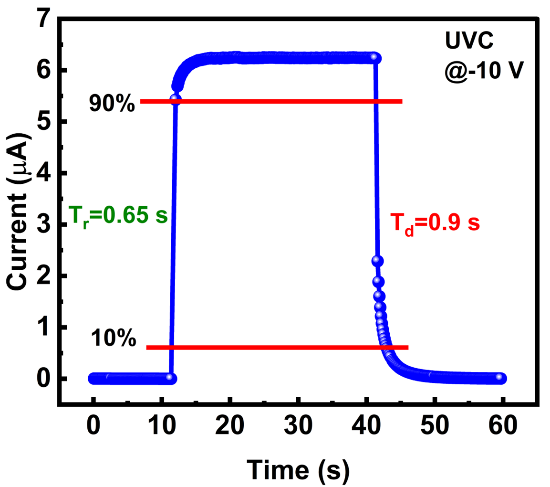
**

**Figure S6.** Single cycle plot of IT at 230 nm to measure the speed of the detector.


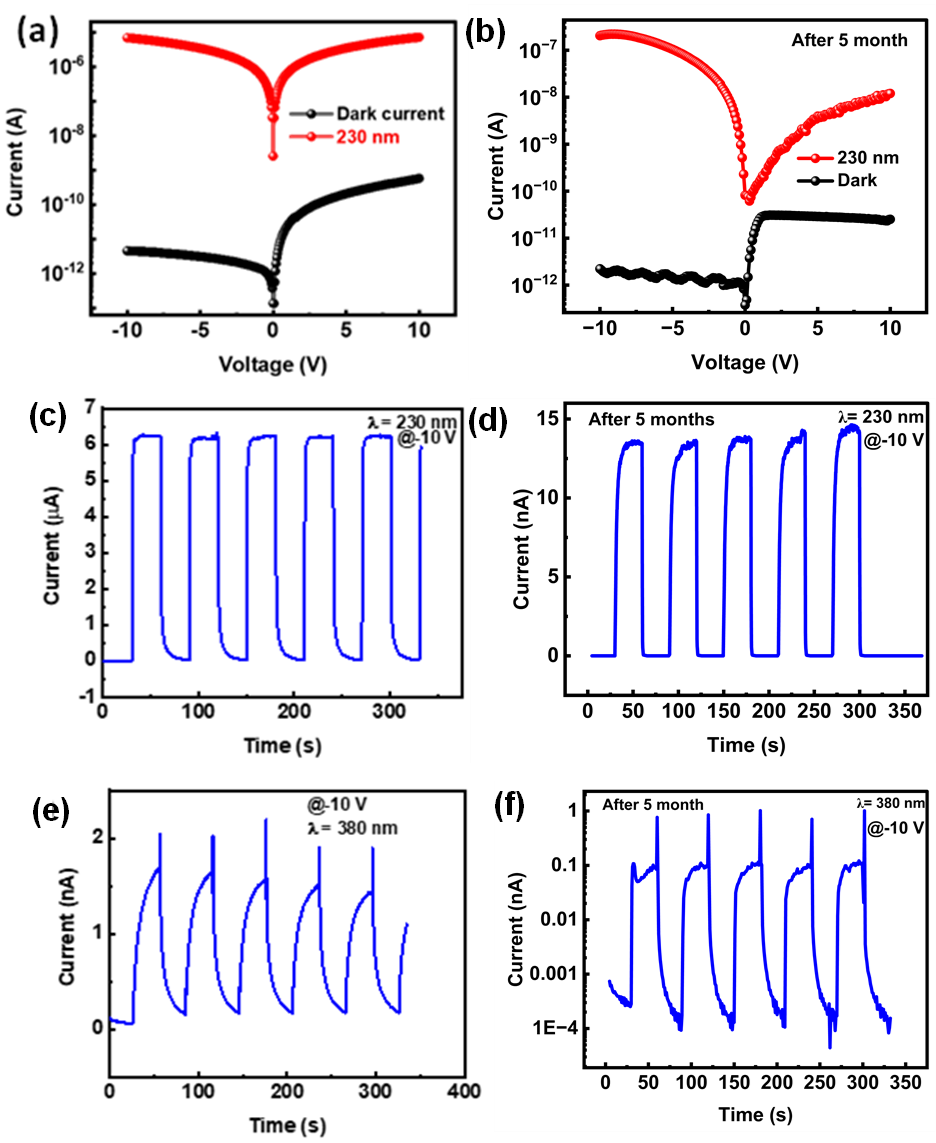


**Figure S7**. Device stability data after 5 months (a) IV for the as fabricated device, (b) IV of the device after the 5 month, (c) cyclic IT response of the device for 230 nm of as fabricated device, (d) cyclic IT response of the device for 230 nm after the 5 months,(e) cyclic IT response of the device for 380 nm of as fabricated device and (f) cyclic IT response of the device for 230 nm after the 5 months.

**Supplementary Note 1: Calculation of energy consumption in forgetting synaptic event**

Characterization limits prevent us from directly measuring energy consumption, but photocurrent response and applied bias can estimate the mathematical formula.

$E=V\times I\times T$ (joule in MKS)

Where, V stands for the applied bias, I minimum synaptic current and T denotes the synaptic event time.

Estimated the values from event in the figure 7 (a) in main manuscript. the forgetting after the 180 s for the pulse of the 5 s. the synaptic event occurred due to the pyrophotoronic effect within the ms: T=10 ms, V=-10 and I= 0.9 nA.

$E=10\times(0.15 nA)\times(10 ms)$ joule

$E=$ 1.5 pJ

Existing optoelectronic synapses: Typically, in the pJ to nJ range.^[1–6]^

Our ZnO/ZnGa_2_O_4_ heterostructure's low operating voltage and strong pyrophototronic effect result in less energy-intensive synaptic events compared to traditional optoelectronic synapses. Many optoelectronic synapses operate in the picojoule to nanojoule range, while biological synapses consume 1–10 fJ each synaptic event. With minimal energy input, the pyrophototronic effect powers our device and improves carrier dynamics.^[1–6]^

**References:**

[1] X. Liu, S. Li, Z. Li, F. Cao, L. Su, D. V. Shtansky, X. Fang, *ACS Appl. Mater. Interfaces* **2022**, *14*, 48936.

[2] W. X. Zhang, H. Li, H. Cong, R. Zhou, Y. Qin, P. Xu, X. Liu, F. Wang, *Curr. Appl. Phys.* **2023**, *48*, 34.

[3] W. Wang, S. Gao, Y. Li, W. Yue, H. Kan, C. Zhang, Z. Lou, L. Wang, G. Shen, *Adv. Funct. Mater.* **2021**, *31*, 1.

[4] L. Liu, Z. Cheng, B. Jiang, Y. Liu, Y. Zhang, F. Yang, J. Wang, X. F. Yu, P. K. Chu, C. Ye, *ACS Appl. Mater. Interfaces* **2021**, *13*, 30797.

[5] F. Cao, E. Hong, Z. Hu, Y. Liu, B. Sun, J. H. He, X. Fang, *Nano Energy* **2024**, *120*, 109135.

[6] X. Li, H. Yu, R. Fang, Y. Lv, Z. Zhou, W. Song, L. Zhang, *IEEE Trans. Electron Devices* **2023**, *70*, 5997.
